# The effect of heat shock protein 90 inhibitors on histone 4 lysine 20 methylation in bladder cancer

**DOI:** 10.17179/excli2018-1807

**Published:** 2019-03-22

**Authors:** Nuran Coban, Nuray Varol

**Affiliations:** 1 Kocatepe University, Faculty of Medicine, Department of Medical Genetics, Afyonkarahisar, Turkey; 2 Afyonkarahisar Health Sciences University, Faculty of Medicine, Department of Medical Genetics, Afyonkarahisar, Turkey

**Keywords:** HSP90, histone lysine methylation modifications, DNA methylation, bladder cancer

## Abstract

Heat shock protein 90 (HSP90), an ATP-dependent molecular chaperone required for the stability and function of numerous oncogenic signaling, is one of the hallmarks of cancer. Recent years, the studies showed that HSP90 plays a pivotal role in epigenetic pathways. Epigenetic regulation plays an important role in the etiology of bladder cancer. The aim of the present study was to investigate the effect of HSP90 proteins on DNA methylation and the levels of inactivated histone methylation markers in bladder cancers. The cytotoxic effect of geldanamycin (GA), a HSP90-specific inhibitor, in human bladder cancer cell line, T24, was studied by using WST1 (both time and dose-dependent), qPCR for the expression aberration of target genes *DNMT1* and *WIF-1* and western blot for the protein levels of DNMT1, Histone H4, Histone 4 lysine monomethylation (H4K20me1), Histone 4 lysine trimethylation (H4K20me3), Akt1, pAkt1 (S473) and Lysine methyltransferase 5C (KMT5C). High-dose GA treatment decreased cell proliferation. After the GA treatment, *DNMT1* decreased at both transcriptional and translational levels due to Akt1 and pAkt1 (S473) inhibition. Following the GA-induced decrease in *DNMT1*, re-expression of *WIF-1* gene was found at mRNA. In addition, the GA treatment resulted in dose- and time-dependent upregulation/downregulation of histone post-translational modifications (H4K20me1 and H4K20me3) and the KMT5C enzyme responsible for these modifications. There was no significant change in the H4 protein level. These findings may offer a new approach for the determination of the molecular effect of HSP90 on epigenetic regulation and the identification of new molecular targets (HSP90 client proteins) for bladder cancer treatment.

## Introduction

Bladder cancer is the ninth most common malignancy worldwide and the second most common type of urological malignancy (Chehab et al., 2015[6]; Wong et al., 2018[35]). Approximately 90 % of bladder cancers are urothelial carcinoma (transitional cell carcinomas) followed by squamous cell carcinoma (5 %) and adenocarcinoma (2 %), all of which are generally treated with surgery (Chehab et al., 2015[6]; Ma et al., 2014[21]). Due to their high prevalence, multiple recurrence, invasive and metastatic character, transitional cell carcinomas are associated with high healthcare costs. Therefore, the effective treatment strategies are vital not only for patients but also healthcare (i.e., to reduce costs) (Chehab et al., 2015[6]; Wong et al., 2018[35]).

Heat shock protein 90 (HSP90) is a key ATP-dependent molecular chaperone associated with the activation and stabilization of more than 200 target (termed as "client") proteins, most of which involve in important cellular functions (e.g., cell proliferation, differentiation, cell cycle, apoptosis and transcriptional regulation) in physiological process (Li et al., 2012[18]; Tatokoro et al., 2015[29]). These client proteins also make a marked contribution to carcinogenesis of bladder cancer. Therefore, HSP90 is considered a potential target in cancer therapy, especially treatment of bladder cancer (Chehab et al., 2015[6]).

HSP90 plays a vital role in epigenetic regulation, as well as maintaining cellular protein homeostasis either via direct or indirect mechanisms. HSP90 acts as a facilitator of histone-DNA binding in HSP90-histone interactions. DNA methyltransferase 1 (DNMT1) is another client protein for HSP90 and responsible for maintenance of DNA methylation. HSP90 inhibition leads to proteolytic degradation of DNMT1 (Isaacs, 2016[10]).

Histone lysine methylation plays a pivotal role in vital functions, including gene regulation, replication, repair, and genome integrity. Histone lysine methyltransferases and demethylases are responsible for maintenance of genome-wide histone lysine modification patterns. There are numerous reports of mutational inactivation or overexpression of these enzymes, leading to misregulation of histone methylation in cancer (Varier and Timmers, 2011[31]; Rose and Klose, 2014[25]). Also, DNA methylation contributes to the maintaining of histone modifications patterns. For example, DNA methylation inhibits H3K4 methylation, and methyl-CpG binding proteins recruit different chromatin-modifying enzymes, including histone lysine methyltransferases and histone deacetylases in heterochromatin structure (Cedar and Bergman, 2009[5]; Rose and Klose, 2014[25]). In addition, H4K20me3 contributes to the maintenance of telomeres, together with Histone 3 lysine 9 trimethylation (H3K9me3) (Balakrishnan and Milavertz, 2010[1]). Loss of H4K20me3 is a hallmark of human cancers, including breast and bladder cancer, and it is associated with telomere lengthening (Balakrishnan and Milavertz, 2010[1]; Schneider et al., 2011[27]; Yokoyama et al., 2014[36]). Suv4-20h2 (as known as KMT5C) is lysine methyltransferase and catalyzes H4K20me3 from SET8 mediated-H4K20me1 (Brustel et al., 2017[4]).

In the present study, first, DNA methyltransferase inhibitor activity of geldanamycin (GA), a HSP90 specific inhibitor, was examined. Second, the effects of HSP90 on the histone 4 (H4) protein level and H4 modifications (H4 Lysine 20 monomethylation [H4K20me1] and H4K20me3) were examined. Finally, aberrations in expressions of enzymes responsible for H4K20me3 modifications following treatment with HSP90 inhibitor in human bladder cancer cells were identified.

## Materials and Methods

### Cell culture and chemicals

The human transitional carcinoma cell line was kindly gifted from Dr. Ece Konac. T24 cells were cultured in McCoy's 5A medium supplemented with 10 % fetal bovine serum (Sigma-Aldrich, Germany) and 100 U/mL penicillin-100 mg/mL streptomycin (Sigma-Aldrich, Germany). Cells were grown in an incubator in 5 % CO2 at 37 °C. Geldanamycin and 17-Dimethylaminoethyl amino-17-demethoxy-geldanamycin (17-DMAG), water-soluble geldanamycin analog are selectively HSP90 inhibitors, and were obtained from Cell Signalling Technology and Sigma-Aldrich, respectively. 

### Cell proliferation assay

The antitumor effect of GA on the viability of T24 cells was determined using cell proliferation reagent WST1 (Roche, Germany). IC_50_ values for GA were determined by treating cells with different concentrations (0-50 µM) of GA. Briefly, in each well of 96-well plates 5 x 10^3^ cells in 100 mL medium were seeded and treated with GA for 24 h. After incubation, WST-1 solution was added to each well and incubated for 4 h. The absorbance was measured at 450 nm in a microplate ELISA reader. The higher and lower administration doses of both GA and 17-DMAG were determined according to the literature.

### Quantitative real-time PCR and Reverse Transcriptase-PCR 

Total RNA was extracted using Qiazol reagent (Qiagen, Germany). Total RNA (1 µg) was reverse-transcribed using RT^2^ HT First Strand kit (Qiagen, Germany) according to manufacturer's instructions. *DNMT1 *and *WIF-1 *mRNA expression levels were measured using quantitative real-time PCR method. Quantitative real-time PCR was performed on RotorGene Q using qPCR SYBR Green Master Mix (Qiagen, Germany). Gene expression levels were normalized to *GAPDH*. It was not possible to perform real-time analysis of *WIF-11 *because the gene was not expressed in the T24 cells. Therefore, we performed reverse transcriptase PCR (RT-PCR) to demonstrate the reactivation of *WIF-1* using the same primer sets used in the real-time PCR analysis. Likewise, the *GAPDH* gene was used as an internal control. The PCR products were analyzed by using 2 % agarose gel electrophoresis.

### Western blotting 

Total protein extractions have been previously described (Varol et al., 2015[32]). Briefly, cells were lysed in RIPA lysis buffer (CST, USA) containing 1 mM PMSF (Sigma-Aldrich, Germany). The samples were centrifuged for 15 min at 13,500 rpm at 4 °C, and the supernatants were collected. Histone proteins were extracted using a histone extraction kit (Abcam, USA) according to manufacturer's protocols. The proteins were quantified using BCA assay kit (Thermo Pierce, USA). Equal amounts of protein were subjected to immunoblotting using the following primary antibodies: HSP90, DNMT1, Akt1, phospho-Akt (Ser473), Histone 4 (H4), mono-methyl-H4 (Lys20) and tri-methyl-H4 (Lys20), β-actin (loading control) (CST, USA) and KMT5C (Abcam, USA) and diluted as 1:1000. The membranes were washed and incubated with appropriate secondary antibodies (1:1000). The proteins were visualized using Lumina Crescendo Western HRP substrate (Millipore, USA).

### Statistical analysis

The WST1, mRNA and proteins results were replicated three times. The statistical significance level in mRNA expression were analyzed using the pairwise fixed reallocation randomization test. The REST software (2009) was used for group-wise comparison and statistical analysis of expression levels.

## Results

### Determination of GA doses

**Figure 1:**
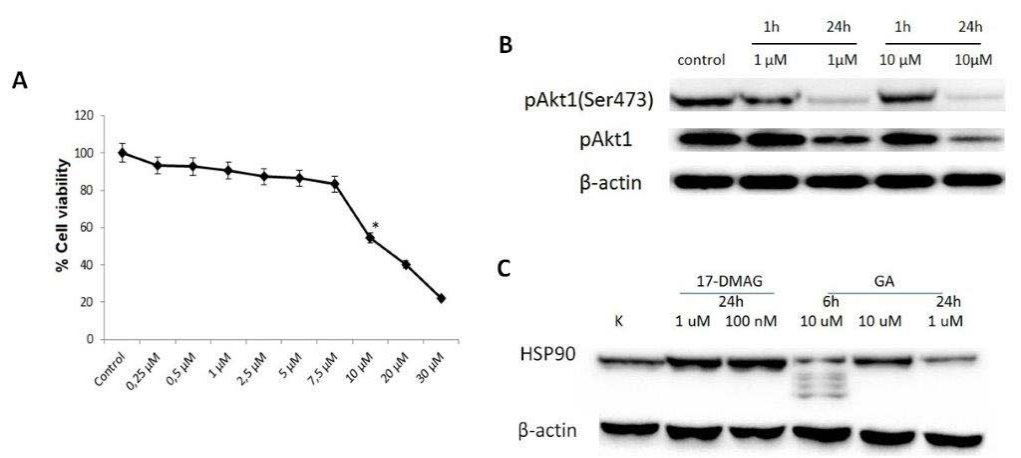
Determination of geldanamycin (GA) doses in T24 cells. A. The effect of the HSP90 inhibitor GA on cell viability. B. Akt1 and pAkt1 (Ser473) protein expression profiles in response to GA treatment for 6 h and 24 h. C. The HSP90 protein level after GA treatment for 6 h and 24 h and 17-DMAG for 24 h.

The optimal dose and timing of GA was determined based on the WST1 assay, and the cells were treated with different concentrations of GA (0-30 µM). The effective dose of GA was 10 µM for 24 h (Figure 1A(Fig. 1)). In the present study, pAkt (Ser473) level did not change significantly following 1 µM GA the for 1 h, whereas the same dose decreased the pAkt (Ser473) level for 24 h (Figure 1B(Fig. 1)). These results are similar to those of Koga et al. (2006[16]), who reported that 1 µM GA was an effective dose for increasing Akt1 and pAkt (Ser473) levels. In the present study, both doses (1 µM and 10 µM) had similar effects on the pAkt (Ser473) level (Figure 1B(Fig. 1)). Thus, 1 µM GA was selected as the low dose.

### The effect of GA on DNA methylation

**Figure 2:**
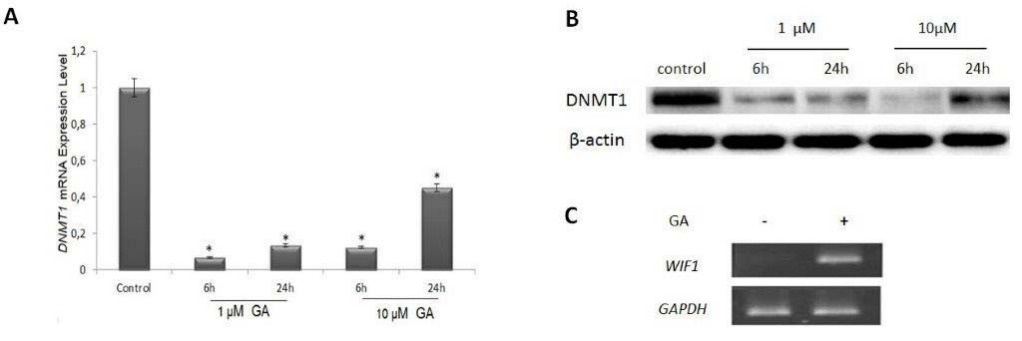
The effect of geldanamycin (GA) on DNA methylation in T24 cells. The distribution of *DNMT1 *mRNA expression (A) and DNMT1 protein levels (B) following treatment with GA. C. Results of *WIF-1 *mRNA expression levels after GA treatment for 24 h.

The expression levels of DNMT1 in T24 cell line were examined using both mRNA and protein analyses. The mRNA expression levels of* DNMT1* decreased in T24 cells treated with 1 µM and 10 µM GA for 6 h and 24 h, as compared with those of untreated control cells as a control (*p<0.05*). In addition, the protein level of DNMT1 decreased in accordance with the mRNA expression of the gene (Figures 2A and 2B(Fig. 2)).

WNT inhibitory factor-1 (WIF-1) is a pivotal antagonist of β-catenin signaling pathway and frequently inactivated via DNA hypermethylation in human cancers. As shown in previous studies, *WIF-1* was hypermethylated and not expressed in T24 cells (Varol et al., 2014[33], 2015[32]). In the present study, after treatment with1 µM and 10 µM GA for 6 h and 24 h, respectively, *WIF-1* was re-expressed in these cells following a reduction of DNMT1 (Figure 2C(Fig. 2)).

### The effect of GA on histone 4 and H4 modifications

**Figure 3:**
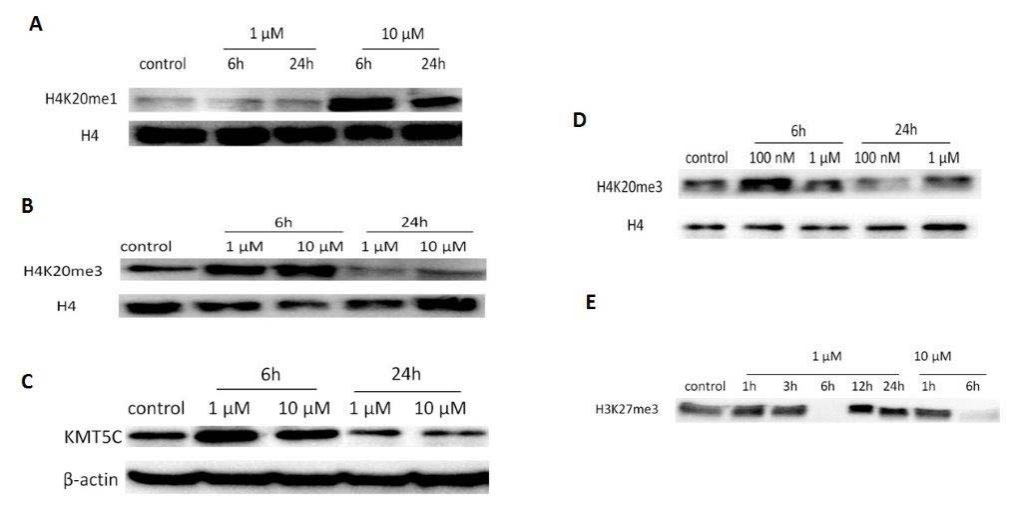
The effect of geldanamycin (GA) on H4 and H4 modifications in T24 cells. A. Protein levels of H4 and H4K20me1. B. Protein levels of H4 and H4K20me3 after GA treatment for 6 h and 24h. C. KMT5C protein expression level following treatment with GA for 6 h and 24 h. D. Protein levels of H4 and H4K20me3 after treatment with 17-DMAG for 6 h and 24 h. E. Protein levels of H3K27me3 after GA treatment for 6 h and 24 h.

The expression level of H4 did not change significantly following the treatment with 1 µM and 10 µM GA. However, H4K20me1 level did not change following 1 µM GA treatment and that of H4K20me1 increased following treatment with 10 µM GA as compared with the levels in control (Figure 3A(Fig. 3)). In contrast to H4K20me1, exposure of bladder cancer cells with GA (1 µM and 10 µM) and 17-DMAG (100 nM) for 6 h increased the H4K20me3 protein level whereas the H4K20me3 protein level treated with GA for 24 h decreased as compared with that in the control (Figure 3B(Fig. 3)). 

In the present study, the KMT5C protein level increased following GA treatment for 6 h and decreased following GA treatment for 24 h (Figure 3C(Fig. 3)). Similar results were observed in the T24 cells treated with 17-DMAG (Figure 3D(Fig. 3)). These results demonstrated that the reduction H4K20me1 level was due to conversion to H4K20me3. 

Additional analysis confirmed the effect of GA treatment for 6 h on histone 3 lysine 27 methylation (H3K27) in the T24 cells. Tri-methylated H3K27 level decreased in dose-independent manner for 6 hours following low-/high-dose GA treatment (Figure 3E(Fig. 3)). Our finding showed that the 6^th^ hour was important for histone 4 lysine 20 methylation in the 24 cells. 

## Discussion

The effectiveness of chemotherapeutics for bladder cancer is frequently reduced due to the development of drug resistance following initially high response rates, and disease recurs in the majority of patients. Effective new agents are urgently needed to improve the survival outcomes and quality of life of bladder cancer patients (Ma et al., 2014[21]). HSP90 is upregulated in cancer cells including lung, breast, bladder cancer (Chehab et al., 2015[6]). Impairment of various signaling pathways contributes significantly to drug resistance. For example, upregulation of PI3K/Akt/mTOR signaling pathway has been implicated in resistance to conventional therapies in many cancer. Many proteins in PI3K/Akt/mTOR signaling pathway are HSP90 client proteins (Safa, 2016[26]).

HSP90 accounts for as much as 1-2 % of total cellular protein. The level of HSP90 increases to 4-6 % under stress-related condition (Whitesell and Lindquist, 2005[34]; Prodromou, 2016[23]). Essential roles of HSPs include maintaining cellular homeostasis, facilitating the adoption of proteins to a mature biologically active conformation, and refolding of misfolding proteins (Ischia and So, 2013[11]). The majority of client proteins are deregulated in human cancers (Whitesell and Lindquist, 2005[34]; Li et al., 2012[18]). Increased ATPase activity or overexpression of HSP90 can trigger the progression of cancer, including bladder cancer (Chehab et al., 2015[6]). Furthermore, HSP90 stabilizes tumorigenic cells by buffering oncogenic mutations. Also, HSP90 prevents misfolding and degradation of oncoproteins and overexpressed or mutated oncoproteins (Li et al., 2012[18]; Ischia and So, 2013[11]; Prodromou, 2016[23]). Many proteins responsible for bladder cancer biology are regulated by HSP90, which promotes oncogenesis (Ischia and So, 2013[11]; Chehab et al., 2015[6]). In addition, the pathological stage and grade are positively correlated with HSP90 overexpression in bladder cancer. In high-grade tumors, HSP90 is expressed on the surface of some cells, suggesting that it may play a role in immune response (Ischia and So, 2013[11]). Therefore, HSP90 may be a potential molecular target for bladder cancer treatment (Karkoulis et al., 2013[13], 2016[14]). Geldanamycin and GA analog 17-DMAG are selective inhibitors for HSP90 function that target the N-terminal domain of HSP90 (Tatokoro et al., 2015[29]; Khandelwal et al., 2016[15]). In this study, HSP90 expression decreased after treatment with low-dose GA for 24 h and high-dose for 6 h, whereas there was no significant change in HSP90 level following 17-DMAG and high-dose GA for 24 h. Our results are consistent with those of Karkoulis et al. (2013[13], 2016[14]). Also, we observed HSP90 proteolytic cleavage exposure in response to high-dose GA for 6 h. HSP90 cleavage of a HSP90 inhibitor was previously described after exposure to high-dose GA (Karkoulis et al., 2016[14]). 

DNA methylation plays an essential role in gene expression and maintaining the epigenetic memory (Bird, 2002[3]; Ghavifekr et al., 2013[8]). DNMT1 is responsible for the maintenance DNA methylation and a HSP90 client protein. DNMT1 is overexpressed and caused inhibition of tumor suppressor genes in human cancer. Inhibition of HSP90 induces proteolytic degradation of DNMT1. EZH2 is also a HSP90 client protein, which interacts with and recruits DNMT1 to target genes. As a result, HSP90 directly and indirectly regulates DNA methylation. In the present study, GA and 17-DMAG treatment of T24 decreased DNMT1 expression at both transcriptional and translational levels, in a dose- and time-independent manner. To evaluate the effect of the GA-induced decrease in DNMT1 expression on DNA methylation, the expression of *WIF-1* is examined*.* *WIF-1* is an important Wnt antagonist. It inhibits Wnt/β-catenin signaling by binding directly to Wnt proteins, and it is frequently hypermethylated in cancer (Varol et al., 2014[33]; Zhang et al., 2014[37]). HSP90 influences Akt/Gsk3β/β-catenin signaling (Liang et al., 2018[20]). Akt1 is an HSP90 client protein and inhibition of HSP90 leads to the reduction of Akt1 expression (Koga et al., 2006[16]; Kurashina et al., 2009[17]). Liang et al. (2018[20]) showed that downregulation of Akt1 by HSP90 inhibition resulted in a decrease in the ratio of pGSK3β(ser9)/GSK3β. A downregulation of Akt1 and an activation of GSK3β were accompanied by the inhibition of β-catenin expression. As reported elsewhere, downregulation of β-catenin resulted in a decrease in DNMT1 expression (Varol et al., 2015[32]). As reported elsewhere, downregulation of β-catenin resulted in a decrease in DNMT1 expression. Previous research also reported that the promoter of the* WIF-1* gene was hypermethylated and that the gene was not expressed in T24 cells (Varol et al., 2014[33], 2015[32]). We demonstrated that the* WIF-1* gene was re-expressed following a reduction in DNMT1 expression after treatment with GA. Thus, HSP90 inhibitors can be used as DNMT inhibitors.

In the regulation of gene expression, DNA methylation is intertwined with histone modification. Recent studies have shed light on the mutual relationship that exists between DNA methylation and histone lysine methylation (Rose and Klose, 2014[25]). DNA methylation has a vital function in gene suppression. It maintains the structural integration of chromatin and regulates histone deacetylation and methylation (e.g., X inactivation and control of cellular development and differentiation) (Baylin, 2005[2]). Methyl-cytosine binding proteins accumulate at methylated CpG sites following DNA methylation, and these proteins recruit chromatin-modifying proteins, including histone deacetylases and lysine methyltransferase in this region, for heterochromatization. Ubiquitin-like with PHD and ring finger domain 1 (UHRF1) localized with PCNA, H3K29me3/2, and hemi-methylated DNA to replication folk, where UHRF1 recruits DNMT1 to maintain DNA methylation on newly synthesized DNA strands (Rose and Klose, 2014[25]).

A second aim of this study was to investigate the effect of HSP90 inhibitors on H4 and lysine 20 modifications (H4K20me1 and H4K20me3). We demonstrated that neither GA nor 17-DMAG influenced H4 protein expression. Histone lysine methylation is a feature of chromatin inheritance (Rivera et al., 2014[24]). H4K20 methylation is related to chromatin silencing. H4K20me3 is found in constitutive heterochromatin regions, including repetitive regions and silenced genes (Füllgrabe et al., 2011[7]). The role of H4K20me1 in the regulation of transcription is complex. Generally, H4K20me1 is found in transcriptionally active regions, but it is associated with chromatin repression in regions repressed during X inactivation. Global histone modification levels (e.g., H3K4me3, H4K20me1, H4K20me, and H4K20me3) are reduced in bladder cancer tissue (Schneider et al., 2011[27]). H4K20me1 is a substrate for the generation of H4K20me3 (Balakrishnan and Milavetz, 2010[1]). In the present study, high-dose GA increased H4K20me1 protein levels in the T24 cells as compared with the levels in untreated T24 cells. H4K20me3 is associated with chromatin repression, specifically in centromeres and telomeres. H4K20me3 is also found in regions of chromatin containing silenced genes. As a result of the absence of H4K20me3 has been positively correlated with the development of cancers (Pogribny et al., 2007[22]; Balakrishnan and Milavetz, 2010[1]). In the present study, the H4K20me3 level increased following the low-/high-dose GA treatment for 6 h and low-dose 17-DMAG for 6 h, whereas it decreased in the cells treated with GA and 17-DMAG for 24 h. Our results are consistent with those of Li et al. (2017[19]). They demonstrated that H4K20me3 and H4K12ac levels increased in a 5637 bladder cancer cell line following treatment with HSP90 inhibitors (AUY922, STAT9090, and SNX-2112).

As reported in recent research, HSP90 interacts with chromatin modifiers. Both KDM3A and KDM4B are HSP90 client proteins and lysine demethylases. Ipenberg et al. (2013[9]) showed that GA-induced inhibition of HSP90 resulted in ubiquitin-dependent proteasomal degradation of KDM4B but not KDM4C. This degradation was accompanied by increased methylation of H3K9. They suggested that HSP90 altered the histone code via regulation of KDM4B stability. To date, no demethylases that act on histone H4K20me3 have been reported (Balakrishnan and Milavetz, 2010[1]). Thus, a third aim of the present study was to identify changes in lysine methyltransferase protein levels following HSP90 inhibition. KMT5C and the tri-methyl transferase H4K20m3 are important for the maintenance of genomic integrity (Shoaib, 2018[28]). KMT5C enzyme mediates H4K20 methylation to H4K20me3 (Jørgensen et al., 2013[12]). We demonstrated that expression of KMT5C increased following treatment with GA for 6 h as a parallel to increased H4K20me3 level. These results show for the first time that KMT5C may be regulated by HSP90. Further studies are required to shed light on the impact of HSP90 on KMT5C. Our results are consistent with those of Tryndyak et al. (2006[30]). They demonstrated that the decrease in trimethylation of H4K20 is accompanied by downregulation of KMT5C histone methyltransferase. However, the loss of KMT5C and consequent decrease in the level of H4K20me3 resulted in telomere lengthening (Balakrishnan and Milavetz, 2010[1]).

As is well known, DNA methylation and histone modifications, particularly lysine methylation, are closely related and dependent on each other. HSP90 plays a vital role in epigenetic regulation, as well as in cellular homeostasis. Aberration of histone lysine methylation frequently occurs in bladder cancer (Chehab et al., 2015[6]). The present study demonstrated that HSP90 regulates not only DNA methylation but also H4 lysine methylation. We suggest that HSP90 inhibition, with consequent impacts on the regulation of DNA methylation and histone modifications, may be an appropriate target for bladder cancer treatment in the future.

## Acknowledgement

This work was supported by The Scientific Research Projects Coordination Unit of Kocatepe University, Project Number: 16.SAG.20.

## Disclosure of conflicting interests

The authors declare that there is no conflict of interest to disclose.
